# TOMOGRAPHIC SARCOPENIA PREDICTS ANASTOMOTIC LEAKS AND LONG-TERM SURVIVAL IN GASTRIC CANCER PATIENTS OPERATED WITH CURATIVE INTENT

**DOI:** 10.1590/0102-672020230002e1723

**Published:** 2023-04-14

**Authors:** Manuel Figueroa-Giralt, Francisca Araya, Andrés Torrealba, José Weisz, Enrique Lanzarini, Maher Musleh, Juan Carlos Molina, Owen Korn, Italo Braghetto, Attila Csendes

**Affiliations:** 1 Universidad de Chile, Clinical Hospital, Surgery Department – Santiago, Chile;; 2 Universidad de Chile, Clinical Hospital, Radiology Department– Santiago, Chile.

**Keywords:** Tomography, Stomach Neoplasms, Fistula, Survival, Sarcopenia, Tomografia, Câncer Gástrico, Fístulas, Sobrevida, Sarcopenia

## Abstract

**BACKGROUND:**

The preoperative nutritional state has prognostic postoperative value. Tomographic density and area of psoas muscle are validated tools for assessing nutritional status. There are few reports assessing the utility of staging tomography in gastric cancer patients in this field.

**AIMS:**

This study aimed to determine the influence of sarcopenia, measured by a preoperative staging computed tomography scan, on postoperative morbimortality and long-term survival in patients operated on for gastric cancer with curative intent.

**METHODS:**

This retrospective study was conducted from 2007 to 2013. The definition of radiological sarcopenia was by measurement of cross-sectional area and density of psoas muscle at the L3 (third lumbar vertebra) level in an axial cut of an abdominopelvic computed tomography scan (in the selection without intravascular contrast media). The software used was OsirixX version 10.0.2, with the tool “propagate segmentation”, and all muscle seen in the image was manually adjusted.

**RESULTS:**

We included 70 patients, 77% men, with a mean cross-sectional in L3 of 16.6 cm2 (standard deviation+6.1) and mean density of psoas muscle in L3 of 36.1 mean muscle density (standard deviation+7.1). Advanced cancers were 86, 28.6% had signet-ring cells, 78.6% required a total gastrectomy, postoperative surgical morbidity and mortality were 22.8 and 2.8%, respectively, and overall 5-year long-term survival was 57.1%. In the multivariate analysis, cross-sectional area failed to predict surgical morbidity (p=0.4) and 5-year long-term survival (p=0.34), while density of psoas muscle was able to predict anastomotic fistulas (p=0.009; OR 0.86; 95%CI 0.76–0.96) and 5-year long-term survival (p=0.04; OR 2.9; 95%CI 1.04–8.15).

**CONCLUSIONS:**

Tomographic diagnosis of sarcopenia from density of psoas muscle can predict anastomotic fistulas and long-term survival in gastric cancer patients treated with curative intent.

## RESUMO

**RACIONAL:**

O estado nutricional pré-operatório tem valor prognóstico pós-operatório. A densidade tomográfica e a área do músculo psoas é uma ferramenta validada para o estado nutricional. Existem poucos estudos avaliando a utilidade da tomografia de estadiamento em pacientes com câncer gástrico neste campo.

**OBJETIVOS:**

Determinar a influência da sarcopenia, medida por tomografia computadorizada de estadiamento pré-operatório, na morbimortalidade pós-operatória e sobrevida em longo prazo em pacientes operados de câncer gástrico com intenção curativa.

**MÉTODOS:**

Estudo retrospectivo de 2007 a 2013. A definição de sarcopenia radiológica foi pela medida da área (PA) e densidade do músculo psoas (PD) a nível de L3 (Terceira vertebra lombar), em um corte axial de tomografia computadorizada abdominopélvica (na seleção sem meio de contraste intravascular). O Software utilizado foi o OsirixX v 10.0.2, com a ferramenta “propagar segmentação”, ajustando manualmente todos os músculos vistos na imagem.

**RESULTADOS:**

Foram incluídos 70 pacientes, 77% homens, PA média em L3: 16,6 cm^2^ (desvio padrão+6,1), PD média em L3: 36,1 mean muscle density (desvio padrão+7,1). Os cânceres avançados foram de 86, 28,6% tinham células em anel de sinete, 78,6% necessitaram de gastrectomia total, a morbidade e mortalidade cirúrgica pós-operatória foi de 22,8 e 2,8%, respectivamente, a sobrevida global de 5 anos a longo prazo (SV5) foi de 57,1%. Na análise multivariada, PA falhou em prever morbidade cirúrgica (p=0,4) e sobrevida global de 5 anos (p=0,34), enquanto PD foi capaz de prever fístulas anastomóticas (p=0,009; OR 0,86; IC95% 0,76–0,96) e SV5 (p=0,04; OR 2,9; IC95% 1,04–8,15).

**CONCLUSÕES:**

O diagnóstico tomográfico de sarcopenia por desvio padrão é capaz de predizer fístulas anastomóticas e sobrevida a longo prazo em pacientes com câncer gástrico tratados com intenção curativa.

## INTRODUCTION

According to GLOBACAN in 2018, gastric cancer is the fifth most common neoplasm and the third most deadly neoplasm in the world^5^. Thanks to surgical technology, oncological drugs, and medical advances in critical care units; surgical morbimortality and long-term survival have improved significantly in the past 50 years^3,10,15,16^. Prognostic factors of postoperative evolution are well described, and the nutritional state has proven to be a relevant short- and long-term independent factors^12,15,18,20^.

Sarcopenia is defined as a loss of muscle strength, quality/quantity, or reduced physical performance^9^; these variables can be assessed in several ways^6,7,13^. Tomographic measurements of sarcopenia using the cross-sectional area (PA) and density (PD) of the psoas muscle are a validated practical approach due to their low cost and frequent use, especially in oncological patients^9^.

The aim of this study was to determine the influence of sarcopenia, measured by a preoperative staging CT (computed tomography) scan, on postoperative morbimortality and long-term survival in patients operated on for gastric cancer with curative intent.

## METHODS

### Design

A retrospective analysis of the oncological database of a Chilean University Hospital (Universidad de Chile Clinical Hospital) from May 2007 to May 2013.

Considering the retrospective nature of the study and the privacy and anonymous analysis of all records, there was no need for institutional IRB approval.

### Patients

All adult patients with gastric adenocarcinoma surgically treated with curative intent with in-hospital tomographic records (ICISview^MR^) of the preoperative staging CT scan were included. Subtotal, total, and extended gastrectomies were included. All the patients were presented to the hospital oncology committee.

Exclusion criteria included gastrectomies due to benign lesions, Stage IV cancers according to the 7th edition TNM classification, R1 resections, type I and II Siewert esophagogastric junction cancers, palliative procedures, complete esophagogastrectomies, and emergency surgeries.

### Definitions

- The TNM classification was standardized using the AJCC 7th edition^18^.
- Surgical mortality was defined as occurring from the moment of surgery up to postoperative day 90.
- Global survival was defined by patients’ discharge from hospitals, eliminating surgical mortality.
- Long-term survival was defined as survival greater than 5 years postoperatively.
- Zero time for determining prognostic association was defined as gastrectomy.

### Computed tomography measurement

**Figure 1:**
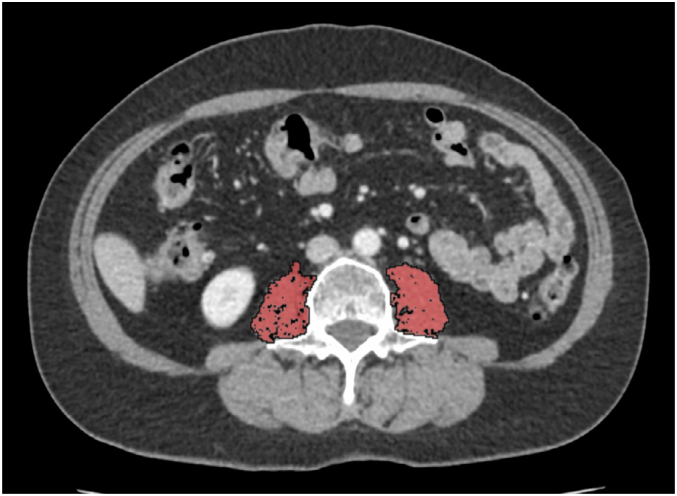
Axial cut of abdominopelvic computed tomography scan without endovascular contrast media. All psoas muscle area is highlighted in red for cross-sectional area and median density measurement.

All preoperative staging CTs were assessed by an expert radiologist with more than 5 years of experience. The mean cross-sectional area (cm^2^) and mean muscle density (HU) were measured at the L3 (third lumbar vertebra) level in an axial cut of an abdominopelvic CT scan (in the selection without intravascular contrast media). The software used was OsirixX version 10.0.2, with the tool “propagate segmentation” adjusting manually all muscles seen in the image (Figure 1).

### Follow-up

The present study has 100% follow-up. The database was completed in a prospective manner; the survival update was carried out annually using the database of our hospital and the Chilean Civil Registry.

### Statistical analysis

The distribution of variables was determined by the Shapiro-Wilk test. In accordance with this test, the continuous variables with parametric or nonparametric distribution were expressed as average and standard deviation (SD) or median and interquartile (IC_25%-75%_) ranges, respectively. The categorical variables were described in percentages. The Fisher's, chi-square, Student's t, and Wilcoxon rank-sum tests were used based on the characteristics and distribution of the variables. For the analytical statistical analysis, the Stata^R^ 14 program was used, and p<0.05 was considered statistically significant. Univariate and multivariate analyses were performed, calculating the odds ratio (OR) with a 95% confidence interval (CI). The Kaplan-Meier and Cox regression were used to calculate survival curves. ROC curves were used to compare prognostic efficacy. The patients signed the informed consent form of the institution.

## RESULTS

A total of 70 patients were included; their median age was 60.5 years (SD±13.6); 77% were men; 83.3% had comorbidities; smoking, hypertension, and diabetes were the most common conditions with 41.4, 34.2, and 17.1%, respectively.

Analysis of the psoas muscle at the L3 level in the staging CT scan showed a mean PD of 36.1 HU (SD±7.1) and a mean PA of 16.6 cm^2^ (SD±6.1).

Notably, 86% of cancers were locally advanced, 58% had intestinal histology according to Lauren's classification, 28.6% had signet ring cells, 78.6% required total gastrectomy, and the mean lymph node dissection was 33.7 (SD±13.9). Staging details are explained in Table 1.

**Table 1:** Distribution of patients according to Americn Joint Committee on Cancer 7th edition^18^.

		n (%)
**T**		
	T1a	8 (11.4)
	T1b	4 (5.7)
	T2	13 (18.6)
	T3	20 (28.5)
	T4a	25 (35.7)
**N**		
	N0	22 (31.4)
	N1	9 (12.9)
	N2	12 (17.1)
	N3a	18 (25.7)
	N3b	9 (12.9)
**TNM Stage (7th ed.)**		
	IA	12 (17.1)
	IB	2 (2.8)
	IIA	9 (12.9)
	IIB	8 (11.4)
	IIIA	12 (17.1)
	IIIB	14 (20.0)
	IIIC	13 (18.6)

Postoperative morbidity and mortality were 22.8 and 2.8%, respectively. The details are explained in Table 2.

**Table 2:** Postoperative surgical adverse event.

Adverse event (*)		n (%)
Surgical		
	Overall	16 (22.8)
	Intra-abdominal abscess	8 (11.4)
	Esophagojejunostomy leak	5 (9.4)
	Duodenal stump leak	2 (2.8)
**Medical**		
	Overall	15 (21.4)
	Respiratory distress	3 (4.3)
	Pleural effusion	2 (2.9)
	Atrial fibrillation	3 (4.3)
	Supraventricular tachycardia	2 (2.9)
	Others	5 (7.1)

* Some patients had ≥2 postoperative morbidities at the same time.

**Figure 2:**
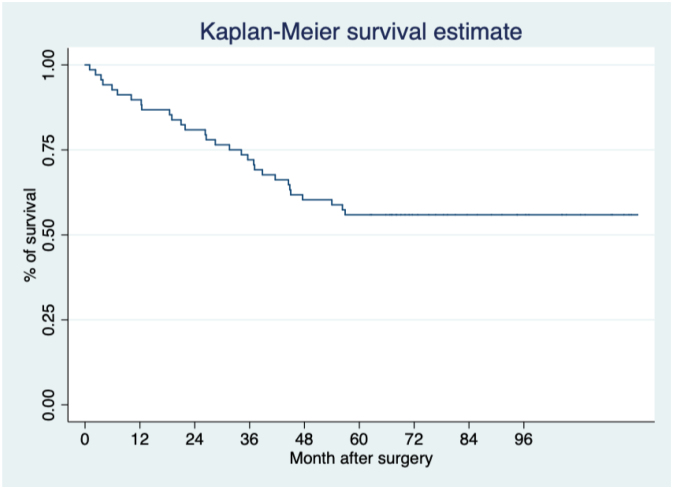
Survival curve of the entire cohort.

Overall long-term survival after five surgeries was achieved in 57.1% of patients (Figure 2).

**Figure 3:**
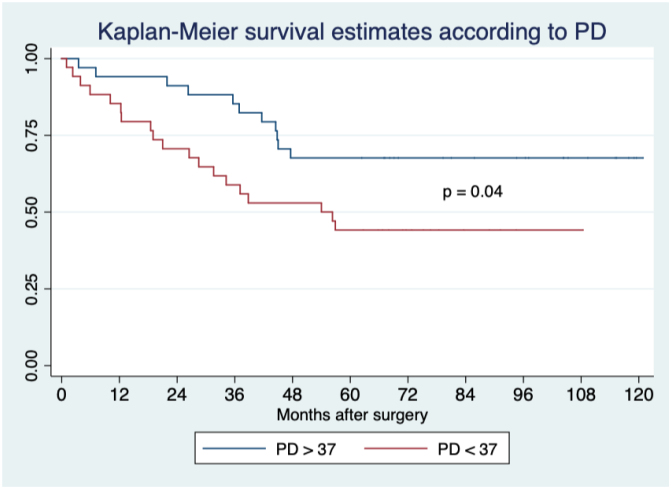
Long-term survival according to density of psoas muscle, the difference between curves is statistically significant with a p=0.04; OR 2.9; 95%CI 1.04–8.15. Density of psoas muscle.

**Figure 4:**
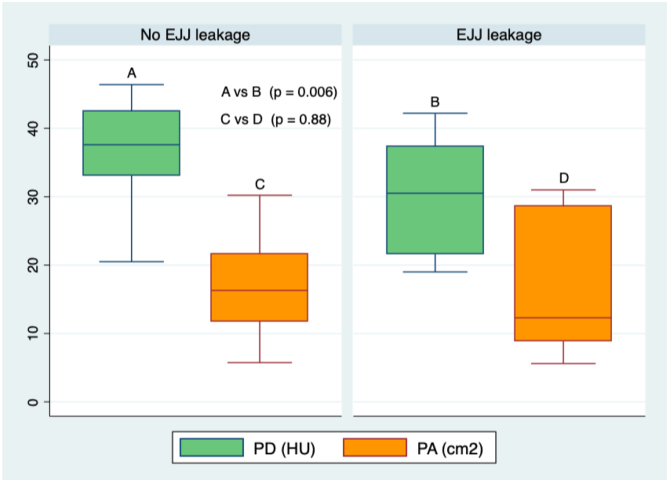
Box plot comparing EJJ leakage according to PD and PA.

EJJ: esophagojejunostomy; PD (HU): mean psoas muscle density; PA: psoas muscle cross-sectional area (cm^2^).

**Figure 5:**
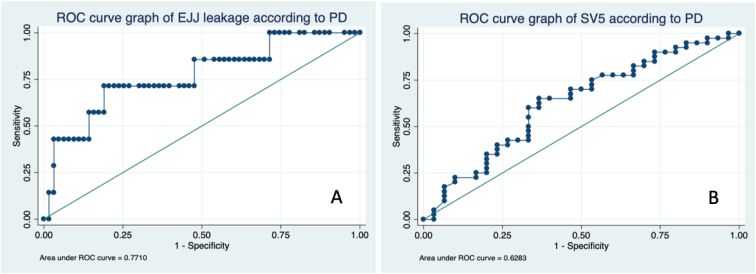
ROC curves.

In the tomographic analysis of sarcopenia, the PA did not achieve statistical significance for any variable measured, whereas PD was associated with esophagojejunostomy (EJJ) leaks and SV5 (Table 3). The optimum stratification analysis for cutoff points of PD in terms of prognostic value in EJJ leaks was 32 HU, with 71.4% sensitivity and 81% specificity, with an ROC area under the curve of 0.77 (RR 4.6; 95%CI 0.56–0.97), while for SV5, it was 37 HU with 63% sensitivity and 63.3% specificity, with an ROC area under the curve of 0.63 (RR 0.94; 95%CI 0.5–0.76). Using this cutoff value, 20 and 50% of our population were in the risk group for EJJ leakage and reduced long-term survival, respectively (Figures 3, 4, and 5).

**Table 3:** Association between different postoperative surgical morbidities and long-term survival, with PA and PD.

	PA	PD
SPOAE	p=0.63	p=0.18
EJJ leaks	p=0.88	p=0.006 (95%CI 2.3–37.9)
Duodenal stump leaks	p=0.82	p=0.18
Surgical mortality	p=0.052	p=0.31
SV5	p=0.45	p=0.02 (95%CI 0.9–0.99)

PA: cross-sectional psoas area; PD: psoas muscle density; SPOAE: surgical postoperative adverse event; EJJ: esophagojejunostomy; SV5: overall 5-year long-term survival.

**A**: EJJ leakage according to PD, the area under the curve is 0.77, **B:** SV5 according to PD, the area under the curve is 0.62.

EJJ: esophagojejunostomy; PD: psoas muscle density; SV5: overall 5-year long-term survival.

The multivariate analysis predicting EJJ leakages showed that PD was the only independent variable with prognostic power (p=0.009; OR 0.86; 95%CI 0.76–0.96). In contrast, analyzing long-term survival, the independent variables were age (p=0.04; OR: 0.65; 95%CI 0.91–0.99), locally advanced stage according to the TNM classification system (p=0.02; OR 0.08; 95%CI 0.009–0.7), and PD (p=0.04; OR 2.9; 95%CI 1.04–8.15) (Table 4).

**Table 4:** Multivariate analysis predicting esophagojejunostomy leakage and long-term survival, according to psoas muscle density and cross-sectional psoas area.

	EJJ leakage			Long-term survival		
	p-value	OR	95%CI	p-value	OR	95%CI
Sex	0.24	–	–	0.39	–	–
Age	0.07	–	–	0.04	0.65	0.91–0.99
Signet ring cells	0.42	–	–	0.21	–	–
Locally advanced*	0.36	–	–	0.02	0.08	0.009–0.7
Peri-lymphatic invasion	0.20	–	–	0.35	–	–
Peri-vascular invasion	0.89	–	–	0.61	–	–
PD	0.009	0.86	0.76–0.96	0.04	2.9	1.04–8.15
PA	0.40	–	–	0.34	–	–

EJJ: esophagojejunostomy; PD: psoas muscle density; PA: psoas muscle cross-sectional area; OR: odds ratio; CI: confidence interval.

* Locally advanced tumors were defined according to TNM stage system 7th edition^18^.

## DISCUSSION

Sarcopenia is a well-known prognostic factor for short- and long-term postoperative outcomes; this pathology has different diagnostic methods and affects a specific group of patients, including oncological ones^9,26^.

Since the introduction of skeletal muscle index (SMI) with CT scan by Prado in 2008^25^, measurement of cross-sectional area and now density of the psoas muscle has proven to predict whole-body muscle accurately independent of body mass index (BMI), achieving short- and long-term prognostic value in different types of cancer^2,14,19,26,27,28^. Recently, a meta-analysis including more than 20,000 patients concluded that preoperative incidence of sarcopenia increases the likelihood of postoperative complications (p<0.001; RR 1.188; 95%CI 1.083–1.303) and overall mortality (p<0.001; HR 1.602; 95%CI 1.369–1.873)^28^.

The use of this practical and accurate tool in gastric cancer patients has been the subject of a few international reports, mostly from Asia^8,21,22,28–31^ and none from Latin American countries to the knowledge of the authors, this report should be the first coming from that region.

Even though diagnostic criteria and cutoff values for sarcopenia vary depending on country, sex, technology [magnetic resonance imaging (MRI) or CT], variables employed [skeletal muscle area (SMA), index (SMI), and mean attenuation (SMRA], the European Working Group on Sarcopenia in Older People recommends the use of two standard deviations below the mean reference value of young, healthy adults^9^. The cutoff value for sarcopenia in the US population according to SMA, SMI, and SMRA are 92.2 cm^2^, 34.4 cm^2^/m^2^, and 34.3 HU in women, and 144.3 cm^2^, 45.4 cm^2^/m^2^, and 38.5 HU in men, respectively^13^. With these parameters, the patients’ rate with sarcopenia in our study was 44.6% (15.0% in women and 57.8% in men), which is higher than the average 34.7% rate in different types of gastrointestinal cancer patients in an international non-Latin American countries^16^ but comparable to other specific gastric cancer reports^21,24,31^. This is an important factor in the discussion; the great heterogeneity of diagnostic criteria and indexes in the Caucasian and Asian literature, sometimes arbitrary, and the lack of this evidence in countries with developing economies, like the present study, makes it difficult to compare results and makes clear the need for scientific evidence.

Postoperative morbidity has been assessed by different studies^8,22,29,30,31^. This is exposed in Table 5. Most of these studies compared SMI with different criteria, and none of them analyzed mean PD; furthermore, most of them do not analyzed long-term survival, except a Chinese prospective study involving 937 patients admitted to radical gastrectomy for gastric cancer whose rate of postoperative morbidity and long-term result were comparable to the present report^29^.

**Table 5:** Studies of gastric patients operated with curative intent, analyzed by tomographic sarcopenia.

Author	n	Year	CT p.	Morbidity (%)	Leaks (%)	SV5 (%)
Wang^29^	255	2015	SMI	43.8 vs 14.3	NS	–
Zhuang^31^	937	2016	SMI	28.5 vs 21.2	–	42.6 vs 69.4
Zhou^30^	240	2017	SMI	49.3 vs 24.6	NS	–
Lou^22^	206	2017	SMI	64.3 vs 23.9	NS	–
Chen^8^	313	2019	SMI	35.1 vs 14.8	NS	–
Figueroa-Giralt^16^	70	2021	PD (HU)	22.8*	15.4 vs 5	44.1 vs 69.4

CT p: tomographic parameter used in the study; SMI: skeletal muscle index measured in cm2/m2; PD(HU): mean psoas muscle density measured; SV5: overall 5-year long-term survival; NS: non-significant. Morbidity (%): All groups of postoperative morbidity (surgical and medical); Leaks (%): Only esophagojejunostomy leaks.

* As the univariate analysis showed that neither psoas muscle cross-sectional area nor psoas muscle density had any prognostic value on morbidity, the rate expresses the frequency of morbidity in the whole group.

Considering the heterogeneity in the diagnostic criteria of radiological sarcopenia, finding the best cutoff points to predict long-term postoperative outcomes is a key factor. In a Japanese^24^ retrospective study of 177 patients with gastric cancer stages II-III admitted to oncological gastrectomy, five cutoff points of SMI (cm^2^/m^2^) were assessed, and the prevalence of sarcopenia varied from 3 to 64%. The best SV5 predicting criteria were achieved with Martin's^23^ cutoff point (SMI 53.0 for BMI>25 or 43.0 for BMI<25 in men, and 41.0 in women), with an SV5 of 48 vs. 68 months (p=0.005; HR 2.0; 95%CI 1.24–3.24). Compared to that report, the present study interestingly achieved a better result with density than with muscle area. These results cannot be totally compared because the abovementioned article did not evaluate PD.

Considering that most patients with gastric cancer will need postoperative adjuvant therapy, it is vital to understand the impact of chemotherapy on the patient's nutrition status^28^. This topic has been studied; a muscle loss ≥9% during chemotherapy is associated with a shorter OS (p<0.001; HR 4.47; 95%CI 2.21−9.05)^4^, which, when added to preoperative sarcopenia, may have a synergic effect. This is especially important in therapeutic strategy if sarcopenia is diagnosed preoperatively, and additional effort in improving performance and nutritional status (prehabilitation) could benefit not only postoperative outcomes but also tolerance to chemotherapy and long-term survival. This hypothesis should be studied in future protocols.

In a palliative context, skeletal sarcopenia, diagnosed by Ct, has proved to be a valuable tool for predicting overall survival. Lee^21^ reported a multivariate analysis of a cohort of 140 consecutive patients who underwent palliative chemotherapy for gastric adenocarcinoma; in that study, sarcopenia was defined as an L3 SMI <49 cm^2^/m^2^ for men and <31 cm^2^/m^2^ for women, using cutoff points specific for the Korean population. Radiological sarcopenia showed poor overall survival (6.8 vs. 10.3 months; p=0.033), which was confirmed in the multivariate analysis (p=0.029; HR 1.51; 95%CI 1.04−2.18); interestingly, no difference in response to chemotherapy was found between patients with and without sarcopenia (p=0.583).

Some articles have analyzed the presence of myokines, which are proteins produced by skeletal muscle with potential anticancer effects. This hypothesis could have a major impact in terms of prehabilitation and postoperative management if a targeted therapy could be found^1,17^.

The present article has the following limitations:

1. Small sample size: The main reason was the lack of CT scans done in our institution; most gastric patients operated on during that period had topographies from different radiological centers. This limitation may have some role in the multivariate analysis and the magnitude of prognostic power.
2. Lack of complementary nutritional studies: Even though tomographic assessment for sarcopenia has been validated, the aim of this study was not to compare this tool with other nutritional diagnostic methods. The additional information would have been valuable to define, whereas the condition of sarcopenia was used not only as the tomographic tool but also as a prognostic variable in this population.
3. Lack of SMI: The database used did not have weight measurements to calculate body surface in more than 80% of patients so that index was impossible to assess.

## CONCLUSION

The findings in this report suggest that PD has a strong prognostic value in predicting leakage of the EJJ and overall 5-year long-term survival, with 32 and 37 HU being the optimal cutoff points, respectively.

More efforts should be needed in Caucasian, North American, and Latin American countries to study tomographic sarcopenia, in order to assess not only the efficacy of prognostic value but also the optimal cutoff points for that specific population.

## References

[1] Aoi W, Naito Y, Takagi T, Tanimura Y, Takanami Y, Kawai Y (2013). A novel myokine, secreted protein acidic and rich in cysteine (SPARC), suppresses colon tumorigenesis via regular exercise. Gut.

[2] Baracos V, Kazemi-Bajestani SM (2013). Clinical outcomes related to muscle mass in humans with cancer and catabolic illnesses. Int J Biochem Cell Biol.

[3] Barchi LC, Ramos MFKP, Dias AR, Forones NM, Carvalho MP, Castro OAP (2021). Brazilian gastric cancer association guidelines (part 2): update on treatment. Arq Bras Cir Dig.

[4] Blauwhoff-Buskermolen S, Versteeg KS, de van der Schueren MA, den Braver NR, Berkhof J, Langius JA (2016). Loss of muscle mass during chemotherapy is predictive for poor survival of patients with metastatic colorectal cancer. J Clin Oncol.

[5] Bray F, Ferlay J, Soerjomataram I, Siegel RL, Torre LA, Jemal A (2018). Global cancer statistics 2018: GLOBOCAN estimates of incidence and mortality worldwide for 36 cancers in 185 countries. CA Cancer J Clin.

[6] Moreno ASC (2015). Anthropometric parameters’ cut-off points for diagnosis of sarcopenia. Nutr Hosp.

[7] Nascimento PRC, Poitras S, Bilodeau M (2018). How do we define and measure sarcopenia? Protocol for a systematic review. Syst Rev.

[8] Chen XY, Li B, Ma BW, Zhang XZ, Chen WZ, Lu LS (2019). Sarcopenia is an effective prognostic indicator of postoperative outcomes in laparoscopic-assisted gastrectomy. Eur J Surg Oncol.

[9] Cruz-Jentoft AJ, Bahat G, Bauer J, Boirie Y, Bruyère O, Cederholm T (2019). Writing group for the European Working Group on Sarcopenia in Older People 2 (EWGSOP2), and the Extended Group for EWGSOP2. Sarcopenia: revised European consensus on definition and diagnosis. Age Ageing.

[10] Csendes JA, Burdiles PP, Braghetto MI, Díaz JJC, Maluenda GF, Korn BO (2006). Evolution of resectability and mortality rates of total and subtotal gastrectomy for gastric cancer. Rev Med Chil.

[11] Rocha IMG, Marcadenti A, Medeiros GOC, Bezerra RA, Rego JFM, Gonzalez MC (2019). Is cachexia associated with chemotherapy toxicities in gastrointestinal cancer patients? A prospective study. J Cachexia Sarcopenia Muscle.

[12] de Las Peñas R, Majem M, Perez-Altozano J, Virizuela JA, Cancer E, Diz P (2019). SEOM clinical guidelines on nutrition in cancer patients. Clin Transl Oncol.

[13] Derstine BA, Holcombe SA, Ross BE, Wang NC, Su GL, Wang SC (2018). Skeletal muscle cutoff values for sarcopenia diagnosis using T10 to L5 measurements in a healthy US population. Sci Rep.

[14] Fearon K, Strasser F, Anker SD, Bosaeus I, Bruera E, Fainsinger RL (2011). Definition and classification of cancer cachexia: an international consensus. Lancet Oncol.

[15] Figueroa-Giralt M, Csendes A, Carrillo K, Danilla S, Lanzarin E (2019). Prognostic factors of long term survival in gastric cancer. Introduction of the new N+/T index. ABCD Arq Bras Cir Dig.

[16] Figueroa-Giralt M, Csendes A, Carrillo K, Danilla S, Lanzarini E, Braghetto I (2019). Introduction of the new lymphoparietal index for gastric cancer patients. ABCD Arq Bras Cir Dig.

[17] Hojman P, Dethlefsen C, Brandt C, Hansen J, Pedersen L, Pedersen BK (2011). Exercise-induced muscle-derived cytokines inhibit mammary cancer cell growth. Am J Physiol Endocrinol Metab.

[18] Jun DH, Kim BJ, Park JH, Kim JG, Chi KC, Park JM (2016). Preoperative body mass index may determine the prognosis of advanced gastric cancer. Nutr Cancer.

[19] Kim EY, Kim YS, Park I, Ahn HK, Cho EK, Jeong YM (2015). Prognostic significance of ct-determined sarcopenia in patients with small-cell lung cancer. J Thorac Oncol.

[20] Lee HH, Park JM, Song KY, Choi MG, Park CH (2016). Survival impact of postoperative body mass index in gastric cancer patients undergoing gastrectomy. Eur J Cancer.

[21] Lee JS, Kim YS, Kim EY, Jin W (2018). Prognostic significance of CT-determined sarcopenia in patients with advanced gastric cancer. PLoS One.

[22] Lou N, Chi CH, Chen XD, Zhou CJ, Wang SL, Zhuang CL (2017). Sarcopenia in overweight and obese patients is a predictive factor for postoperative complication in gastric cancer: A prospective study. Eur J Surg Oncol.

[23] Martin L, Birdsell L, Macdonald N, Reiman T, Clandinin MT, McCargar LJ (2013). Cancer cachexia in the age of obesity: skeletal muscle depletion is a powerful prognostic factor, independent of body mass index. J Clin Oncol.

[24] Nishigori T, Tsunoda S, Obama K, Hisamori S, Hashimoto K, Itatani Y (2018). Optimal cutoff values of skeletal muscle index to define sarcopenia for prediction of survival in patients with advanced gastric cancer. Ann Surg Oncol.

[25] Prado CM, Lieffers JR, McCargar LJ, Reiman T, Sawyer MB, Martin L (2008). Prevalence and clinical implications of sarcopenic obesity in patients with solid tumours of the respiratory and gastrointestinal tracts: a population-based study. Lancet Oncol.

[26] Rodriguez T, Moreno N, Abedrapo M, Bocic G, Azolas R, LLanos JL (2019). Predictive value of sarcopenia for anastomotic dehiscense in colon cancer surgery. Rev Cir.

[27] Sakamoto E, Ramos MFKP, Pereira MA, Dias AR, Ribeiro U, Zilberstein B (2022). Staging laparoscopy is still a valuable tool for optimal gastric cancer management. ABCD Arq Bras Cir Dig.

[28] Su H, Ruan J, Chen T, Lin E, Shi L (2019). CT-assessed sarcopenia is a predictive factor for both long-term and short-term outcomes in gastrointestinal oncology patients: a systematic review and meta-analysis. Cancer Imaging.

[29] Wang SL, Zhuang CL, Huang DD, Pang WY, Lou N, Chen FF (2016). Sarcopenia adversely impacts postoperative clinical outcomes following gastrectomy in patients with gastric cancer: a prospective study. Ann Surg Oncol.

[30] Zhou CJ, Zhang FM, Zhang FY, Yu Z, Chen XL, Shen X (2017). Sarcopenia: a new predictor of postoperative complications for elderly gastric cancer patients who underwent radical gastrectomy. J Surg Res.

[31] Zhuang CL, Huang DD, Pang WY, Zhou CJ, Wang SL, Lou N (2016). Sarcopenia is an independent predictor of severe postoperative complications and long-term survival after radical gastrectomy for gastric cancer: analysis from a large-scale cohort. Medicine (Baltimore).

